# Old Proverbs in New Skins – An fMRI Study on Defamiliarization

**DOI:** 10.3389/fpsyg.2012.00204

**Published:** 2012-07-04

**Authors:** Isabel C. Bohrn, Ulrike Altmann, Oliver Lubrich, Winfried Menninghaus, Arthur M. Jacobs

**Affiliations:** ^1^Department of Education and Psychology, Freie Universität BerlinBerlin, Germany; ^2^Cluster of Excellence “Languages of Emotion,” Freie Universität BerlinBerlin, Germany; ^3^Institut für Germanistik, Universität BernBerne, Switzerland; ^4^Department of Comparative Literature, Freie Universität BerlinBerlin, Germany; ^5^Dahlem Institute for Neuroimaging of EmotionBerlin, Germany

**Keywords:** reading, familiarity, fMRI, esthetics, foregrounding, proverbs, literature

## Abstract

We investigated how processing fluency and defamiliarization (the art of rendering familiar notions unfamiliar) contribute to the affective and esthetic processing of reading in an event-related functional magnetic-resonance-imaging experiment. We compared the neural correlates of processing (a) familiar German proverbs, (b) unfamiliar proverbs, (c) defamiliarized variations with altered content relative to the original proverb (proverb-variants), (d) defamiliarized versions with unexpected wording but the same content as the original proverb (proverb-substitutions), and (e) non-rhetorical sentences. Here, we demonstrate that defamiliarization is an effective way of guiding attention, but that the degree of affective involvement depends on the type of defamiliarization: enhanced activation in affect-related regions (orbito-frontal cortex, medPFC) was found only if defamiliarization altered the content of the original proverb. Defamiliarization on the level of wording was associated with attention processes and error monitoring. Although proverb-variants evoked activation in affect-related regions, familiar proverbs received the highest beauty ratings.

## Introduction

The emerging field of neuroesthetics is marked by a lack of neuroimaging studies regarding the esthetic perception of literature (Schrott and Jacobs, 2011). In this paper, we will use the term “literature” in a broad sense including any text that has the potential to elicit an esthetic response either triggered by certain features of the text itself, such as rhetorical figures, or by external framing, for instance by the label “novel” on the front cover (cf. Bleich, 1978; Schrott and Jacobs, 2011). Likewise, we use the term “esthetics” in the broad sense of neuroesthetics “to encompass the perception, production, and response to art, as well as interactions with objects and scenes that evoke an intense feeling, often of pleasure” (Chatterjee, 2011, p. 53). This paper aims to investigate how feelings of pleasure, so familiar to everyone who enjoys a good book or poem, can arise from a task such as reading, which is primarily based on a number of cognitive skills. The key concept of *defamiliarization* refers to the use of artistic techniques in order to turn something familiar into something that appears unfamiliar or strange. Specifically, we investigated to which extent defamiliarization (Miall and Kuiken, 1994) contributes to the affective and esthetic perception of literature by making it harder to process. In the following, we will introduce three theoretical frameworks of how cognitive load potentially relates to affective responses and preference judgments.

### Hedonic fluency hypothesis

A number of theories predict a higher preference for familiar and conventional stimuli over novel and unfamiliar ones: describing the mere-exposure effect, Zajonc (1968) claimed that mere repeated exposure to a stimulus is sufficient for people to develop a positive attitude toward it. In a seminal study, Zajonc (1968) demonstrated a positive correlation between word frequency and affective connotation. Such a positive correlation between exposure frequency and preference has been replicated several times for a number of different stimulus categories and can be regarded as a robust effect, although it is influenced by certain variables such as the number of repetitions, initial familiarity etc. (Bornstein, 1989). The preference-for-prototypes effect (Martindale and Moore, 1988; Martindale et al., 1990; Winkielman et al., 2006) shows that more typical members of a category are frequently preferred over less typical members. Both effects are probably driven by the force of perceptual fluency, the ease by which a sensory input can be processed. The hedonic fluency hypothesis states that simply because a stimulus with a high familiarity/typicality/expectedness/exposure is processed faster than a novel or unknown stimulus, it is accompanied by a positive affective evaluation (Reber et al., 1998, 2004; Winkielman and Cacioppo, 2001). In literature, processing fluency could, for instance, be modulated through the use of stylistic devices that regulate the cognitive processing demand (e.g., formulaic expressions, repetition figures). Fluency and familiarity evaluation are automatic processes that can influence the esthetic judgment prior to conscious processing (Kunst-Wilson and Zajonc, 1980; Leder et al., 2004; Kuchinke et al., 2009). Applying the hedonic fluency hypothesis to the process of reading, the prediction follows that easy-to-read text is preferred over more difficult-to-read text. Indeed, subtle semantic coherence of words can increase the experience of hedonic fluency (Topolinski et al., 2009), and comparable effects have been shown for rhetorical devices, especially repetition figures such as rhyme (McGlone and Tofighbakhsh, 2000), alliteration (Lea et al., 2008), and parallelistic syntactic structures (Sturt et al., 2010). The main prediction following the hedonic fluency hypothesis would be that text with low cognitive processing demand is preferred over more difficult-to-read text.

### Foregrounding hypothesis

Defamiliarization, achieved through “the novelty of an unusual linguistic variation” (Miall and Kuiken, 1994, p. 391), is a very influential concept for twentieth century art, impacting for instance film, theater, visual arts, and literature. Already the Russian Formalists and Czech Structuralists (Mukarovský, 1964; Shklovsky, 1998) claimed that “the technique of art is to make objects ‘unfamiliar,’ to make forms difficult, to increase the difficulty, and length of perception because the process of perception is an esthetic end in itself and must be prolonged” (Shklovsky, 1998, p. 18). Following their ideas, the concept of foregrounding is used “to indicate the (psycholinguistic) processes by which – during the reading act – something may be given special prominence” (Van Peer and Hakemulder, 2006). The theory of foregrounding is based on principles of cognitive psychology and the empirical study of literature, suggesting that certain linguistic devices on the phonetic, syntactic, or semantic level can be used to defamiliarize the reading experience and thereby slow down the automatic reading process even in skilled readers. Empirical evidence comes from behavioral studies (van Peer, 1986; Miall and Kuiken, 1994; Hanauer, 1998; Hakemulder, 2004), in which participants gave stronger affect and “strikingness” ratings for those literary texts that took longer to read due to a high density of “foregrounding” features (i.e., stylistic elements). According to foregrounding theory (Miall and Kuiken, 1994), reading times increase with higher density of foregrounding devices because stylistic variation increases text complexity. As complexity is a diffuse concept, predictability could serve as a simpler, quantifiable, moderating variable. There is sound empirical evidence that the predictability of words in a sentence context affects eye movements, reading times, and brain potentials (McDonald and Shillcock, 2003; Rayner et al., 2004; Frisson et al., 2005; Dambacher et al., 2006, 2009; Dambacher and Kliegl, 2007). Unexpected words slow down the speed of reading and cause characteristic event-related brain potentials (Kutas and Hillyard, 1980). Apart from such cognitive effects, discrepancy can also raise physiological arousal (MacDowell and Mandler, 1989), trigger appraisal processes (Scherer, 2001), and be accompanied by interest or surprise (Silvia, 2008). When readers are confronted with novel, unexpected elements in a text, they usually react emotionally with curiosity and dishabituation (Oatley, 1994). High defamiliarization by means of stylistic variation lowers the predictability of single words in a text and promotes the esthetic perception of poetry and literature (Miall and Kuiken, 1994; Hanauer, 1998). In summary, foregrounding theory predicts that a text which is highly defamiliarized through the use of stylistic elements will be preferred over text which is easier to read, because it is processed in a more affective manner.

### Optimal innovation hypothesis

An alternative to the theories described above is provided by the *optimal innovation hypothesis* (Giora et al., 2004). A phrase that elicits a salient response by carrying familiar elements while at the same time eliciting a non-salient, novel response (e.g., weapons of *mass distraction*), should be more pleasurable than an all too easily processed, conventional phrase (e.g., weapons of *mass destruction*), which only elicits a salient response. It should equally be more pleasurable than a similar-sounding, novel phrase that only elicits a non-salient response (e.g., weapons of *glass deduction*). For a response to be “novel” in the sense of Giora and colleagues, it has to bring forward a “discretely different conceptual meaning than the one activated by the familiar original from which it stems” (Giora et al., 2004, p. 117). As difficult as it is to define what makes an “optimally innovative stimulus” (which is further complicated by interindividual differences), twists of conventional expressions, so-called “proverb-variants” (e.g., Absence makes the heart go wander), are a common rhetorical device in journalism, advertisement, song lyrics, or catch-phrases, to raise attention and often to create ironic effects (Mieder, 2008). The ability creatively to transform figurative language and to create novel metaphors and figurative expressions is one of the final abilities acquired in the process of language learning, as it requires the ability to reflect on language (Levorato and Cacciari, 2002), and is used as a measure of verbal creativity (Bechtereva et al., 2007; Fink et al., 2007). The fluency hypothesis and the theory of foregrounding/defamiliarization postulate a linear relationship between cognitive processing effort and pleasure. The optimal innovation hypothesis, on the contrary, states a non-linear relationship by predicting that the most pleasing text will offer neither a maximum ease of processing fluency nor a maximum degree of defamiliarization, but will provide an optimal combination of both dimensions.

### Present experiment

The present experiment investigated how processing fluency and defamiliarization modulate the affective and esthetic perception of proverbs by manipulating proverb familiarity and introducing two different types of defamiliarized proverb-variants. Explicit reader-responses and changes in the regional cerebral blood flow, measured by functional magnetic-resonance-imaging (fMRI) were analyzed. While not intuitively associated with the term “literature” by many people, proverbs turned out to be adequate stimuli for this purpose of interdisciplinary research. Short enough to fulfill multiple experimental requirements, they are at the same time complex psycholinguistic stimuli that people encounter in real life, and that are applied for clinical diagnosis (Gibbs and Beitel, 1995; for a comprehensive review see Thoma and Daum, 2006).The five experimental conditions (exemplified in Table 1 and described in greater detail in the Materials and Methods) were

**Table 1:** **Condition overview**.

Familiarity	Rhetorical	Condition	Examples (*English Translation*)
High	Yes	Familiar proverbs	**Wissen** ist Macht (**Knowledge** *is power*)
			Wer **wagt**, gewinnt (*Who* **dares**, *wins*)
			Reden ist Silber, **Schweigen** ist Gold (*Talk is silver*, **silence** *is golden*)
			Zeit **ist** Geld (*Time* **is** *money*)
			**Ende** gut alles gut (*All’s well*, *that* **ends** *well*)
Low	Yes	Unfamiliar proverbs	Bruderzwist gar heftig ist (*Fraternal strife is fierce*)
			Jahre lehren mehr als Bücher (*Years teach more than books*)
			Einfalt hat schöne Gestalt (*Naivity comes in good shape*)
			Kleiner Mann, großes Herz (*Small man*, *big heart*)
			Nicht alle Wolken regnen (*Not every cloud brings rain*)
Defamiliarized	Yes	Proverb-substitutions	**Kenntnis** ist Macht (**Information** *is power*)
			Wer **riskiert**, gewinnt (*Who* **risks**, *wins*)
			Reden ist Silber, **Stille** ist Gold (*Talk is silver*, **stillness** *is golden*)
			Zeit **bedeutet** Geld (*Time* **means** *money*)
			**Schluss** gut, alles gut (*All’s well*, *that* **finishes** *well*)
Defamiliarized	Yes	Proverb-variants	**Gewissen** ist Macht (**Conscience** *is power*)
			Wer **fragt**, gewinnt (*Who* **asks**, *wins*)
			Reden ist Silber, **Helfen** ist Gold (*Talk is silver*, **helping** *is golden*)
			Zeit **frisst** Geld (*Time* **eats** *money*)
			**Rente** gut, alles gut (*All’s well*, *that* **pays** *well*)
Medium	No	Non-rhetorical sentences	Fleiß führt oft zum Erfolg (*Effort often leads to success*)
			Viele Beziehungen halten nicht (*Many relationships do not last*)
			Etwas Sport ist gesund (*Modest exercise is healthy*)
			Lachen entspannt im Alltag (*A laugh relaxes everyday life*)
			Man soll das Leben heiter verbringen (*One should live cheerfully*)

*Note. Critical words that differ between familiar proverbs, proverb-substitutions, and proverb-variants are written in bold*.

(a) familiar proverbs (e.g., *Rome was not* *built in a day*)
(b) unfamiliar proverbs (e.g., *Not every cloud rains*)
  Similar to McGlone et al. (1994), two versions of manipulated proverbs, based on the template of a familiar proverb were created:
(c) proverb-substitutions: defamiliarized versions of the familiar proverbs in which one word was substituted by a close synonym, thereby violating the form but not changing the content (e.g., *Rome was not* *erected in a day*). This version was considered relatively low innovative.
(d) proverb-variants: defamiliarized versions of the familiar proverbs in which by substitution of a single word the central concept of the familiar proverb was changed (e.g., *Rome was not* *destroyed in a day*). This version was considered relatively high innovative.
(e) literal, non-rhetorical sentences that served as a baseline condition (e.g., *Salt makes better taste*).

### Explicit esthetic judgments

The three theoretical frameworks described so far make different predictions regarding explicit esthetic judgments (i.e., ratings of “beauty” given by participants after the fMRI experiment). Given that familiar proverbs are the easiest condition to process, according to the hedonic fluency hypothesis one can predict a linear relationship between stimulus complexity (which affects processing fluency) and beauty ratings in a way that familiar proverbs will be favored, followed by the simple literal sentence condition (e) and the defamiliarized conditions (c, d), while the most difficult-to-process, unfamiliar proverbs should score the worst. Galak and Nelson (2011) demonstrated that when reading for enjoyment, readers prefer text that they can read fluently and a positive linear relationship between processing fluency and liking has been described before (Reber et al., 1998). Foregrounding theory makes just the opposite prediction: highly foregrounded unfamiliar proverbs would be preferred to proverb-variants (d) and proverb-substitutions (c), followed by familiar proverbs. The literal condition (e), lacking any foregrounding elements, should receive the lowest esthetic appreciation. Third, the optimal innovation hypothesis predicts a non-linear relationship between cognitive fluency and pleasure and thus qualitative differences between the two defamiliarized versions: Proverb-variants should be favored over proverb-substitutions, because only the former fulfill the criteria of being “optimally innovative” (Giora et al., 2004). The latter, being also defamiliarized versions of the original proverbs but maintaining their central concepts, meet the criterion of defamiliarization, but are not “optimally innovative.” Recognizing the original proverb elicits a salient response, while at the same time the unexpected word triggers a non-salient response. Following from the optimal innovation hypothesis, proverb-variants (d) should be favored over all other conditions.

### Predicted neural correlates

While the three theoretical frameworks described above make predictions about explicit reader response, these theories clearly are based on behavioral experiments and are mute regarding the brain. However, a positive explicit esthetic judgment could potentially be reflected in activation of affect- and reward-related brain regions. Assuming that familiarity and defamiliarization modulate explicit reader response, we hypothesized that these two dimensions would also modulate the involvement of the reward system (Kringelbach et al., 2008), and regions formerly found for affective processing of single words. A recent review by Citron (2012) highlights the influence of emotion variables on written word processing. Based on previous neuroimaging studies, we expected to find activity related to familiarity and defamiliarization in brain regions such as the left amygdala (Landis, 2006), extrastriatal visual regions (Herbert et al., 2009), the striatal region (Hamann and Mao, 2002), the left orbito-frontal cortex (OFC), the bilateral inferior frontal gyrus (IFG), and the superior frontal and middle temporal gyrus (SFG/MTG; Kuchinke et al., 2005; Jacobs, 2011).

To investigate effects of *familiarity* independently from rhetorical foregrounding, we contrasted the neural correlates of reading familiar proverbs against reading unfamiliar proverbs. A stronger engagement of affect-related regions during the reading of familiar proverbs would support the hedonic fluency hypothesis, while a similar activation during the reading of unfamiliar proverbs would support the foregrounding theory.

To investigate effects of *defamiliarization* we contrasted the neural correlates of reading the two types of defamiliarized proverbs (“optimally innovative” proverb-variants and proverb-substitutions) both with familiar proverbs and with each other. If both defamiliarized conditions activated affect-related regions to a similar degree, this would be in line with the foregrounding theory. Differences in the intensity of activation between proverb-variants and proverb-substitutions would support the *optimal innovation hypothesis*, whereas stronger affective involvement for familiar proverbs would be in line with the hedonic fluency hypothesis.

Additionally, we assumed that foregrounding and defamiliarization would be correlated with increased attention demands. We expected activation of the bilateral frontoparietal attention network, covering both inferior frontal gyri and the inferior parietal lobes (Corbetta and Shulman, 2002). Familiar proverbs and non-rhetorical sentences were expected to serve as background conditions that were relatively easy to process. Thus, for the familiar proverbs and non-rhetorical sentences we predicted relatively stronger activity within the default mode network (Buckner et al., 2008), which is usually involved in self-referential thinking during resting state.

## Materials and Methods

### Participants

Twenty-six healthy participants underwent the fMRI study (mean age 25 years, range 20–45; 13 female, 13 male). Informed consent was obtained from all participants and the experiment was approved by the local ethics committee (Charité, Berlin). All participants were native German speakers, right-handed as determined by the Edinburgh handedness inventory (Oldfield, 1971), had normal or corrected-to-normal vision, and neither obvious reading deficits (assessed with the SLS – Salzburger Lesescreening; unpublished version for adults), nor a history of neuropsychiatric disorders or psychoactive medication.

### Stimuli

In total, five different categories of stimuli were shown, each comprising 40 items. To investigate effects of familiarity, (a) familiar proverbs, frequently used in German, and (b) unfamiliar German proverbs were collected. Familiar proverbs are conventionalized and therefore rather easy to process. They are “prefabricated: that is, stored and retrieved as a whole from memory at the time of use” (Wray, 2002, p. 9), and are therefore read faster than novel phrases (Cacciari and Tabossi, 1988; Tabossi et al., 2009). To investigate effects of defamiliarization, two types of defamiliarized proverbs were created: (c) proverb-variants, in which the content of a familiar proverb was twisted by replacing a single word. Proverb-variants can thus be considered as defamiliarization of common proverbs mainly on the semantic level. In contrast, (d) proverb-substitutions were created by replacing a single word of a familiar proverb with a synonym, thereby preserving the original meaning as far as possible, but violating the conventional form. Proverb-substitutions can be considered as a type of defamiliarization affecting mainly the level of style and wording. To have a high-level baseline, (e) 40 non-rhetorical sentences, which lacked proverb-characteristic stylistic features and had a valid literal interpretation, served as a control condition (Table 1; see Appendix for a complete list of stimuli). Non-rhetorical sentences were carefully chosen to match the familiar proverbs and unfamiliar proverbs regarding topics (simple statements about folk psychology and world-knowledge) but importantly, they did not correspond to specific proverbs and they lacked proverb-characteristic rhetorical features. All other conditions were matched in number and type of rhetorical features (i.e., phonological similarities such as rhyme/alliteration, meter, parallelism, and ellipses).

All conditions were matched for important lexical parameters such as number of words, number of syllables, and mean word frequency taken from the Wortschatz Lexikon of the University of Leipzig^1^. Google counts dating from July 2009 were used as an approximation of the whole item’s frequency and are reported in Table 2 with all other lexical parameters. The frequency measures indicate that in real life the familiar proverbs occur far more frequently than all other conditions (all *p*s < 0.001) and that the non-rhetorical control items occur the least frequently (all *p*s < 0.05).

**Table 2:** **Formal stimulus characteristics**.

	Words		Digits		Syllables		Word frequency^1^		Stylistic features		Google counts^1^	
	*M*	SD	*M*	SD	*M*	SD	*M*	SD	*M*	SD	*M*	SD
Familiar	5.08	1.73	29.28	9.36	7.50	2.60	3.79	0.51	2.35	1.00	9.58	2.76
Unfamiliar	5.08	1.97	30.20	9.42	7.85	2.58	3.62	0.56	2.30	1.07	1.68	1.18
Variant	5.08	1.73	29.50	9.13	7.58	2.44	3.74	0.58	2.18	1.06	1.74	2.86
Substitution	5.08	1.73	30.28	8.48	7.73	2.45	3.72	0.55	2.10	1.06	0.94	1.70
Non-rhetorical	5.33	0.89	32.03	4.15	8.38	1.44	3.77	0.72	–	–	0.12	0.41
Significances	n.s.		n.s.		n.s.		n.s.		n.s.		*F*(4,195) = 145.47, *p* < 0.001	

*^1^Mean logarithmic frequency*.

#### Pretests

Familiar and unfamiliar proverbs were selected according to dichotomous familiarity judgments (known/unknown) that 14 participants had given for each item of a pool of 800 German proverbs and aphorisms collected from proverb dictionaries and online databases. Unfamiliar proverbs had been judged as “unknown” and familiar proverbs had been judged as “known” by all 14 participants. Twenty-five participants (12 female, 13 male) not involved in the main fMRI experiment or any other pretest rated the stimulus set regarding arousal and valence using the SAM instrument (Bradley and Lang, 1994). No significant differences in arousal or valence ratings were found. Another sample of 29 (19 female, 10 male) participants not involved in the main fMRI experiment or any other pretest rated the stimulus set on inventiveness using a seven-point Likert scale. In line with classical rhetorical theory of wit and wordplay (Cicero, De oratore, II.216–290), all rhetorical conditions (familiar proverbs, unfamiliar proverbs, proverb-substitutions and proverb-variants) were rated as significantly more inventive than the non-rhetorical sentences. Among the rhetorical conditions, proverb-substitutions, in which the original proverb’s wording was violated, but not the content level, were rated as significantly less inventive than the others. See Table 3 for a list of Means and SDs of all pretest ratings.

**Table 3:** **Pretest ratings**.

	Valence		Arousal		Inventiveness^1^	
	*M*	SD	*M*	SD	*M*	SD
Familiar proverbs	4.98	1.14	5.44	0.66	3.58	1.34
Unfamiliar proverbs	4.71	0.99	4.88	0.73	3.42	0.81
Variants	4.87	1.09	4.97	0.55	3.47	0.83
Substitutions	4.65	0.95	5.04	0.61	2.94*	0.89
Non-rhetorical	4.74	1.45	5.10	0.74	2.44***	1.00
Significances	n.s.		n.s.		*F*(2.4,68.2) = 15.04, *p* < 0.001, ${\text{η}}_{p}^{2}=0.35$	

*^1^Non-rhetorical sentences received significantly lower inventiveness ratings than familiar, unfamiliar, and proverb-variants (*p* < 0.001), and significantly lower inventiveness ratings than proverb-substitutions (*p* < 0.05). Proverb-substitutions received significantly lower inventiveness ratings than familiar, unfamiliar, and proverb-variants (*p* < 0.01)*.

*Significantly different from all other conditions at p  < 0.05.

***Significantly different from all other conditions at *p* < 0.001.

### Task design

**Figure 1:**
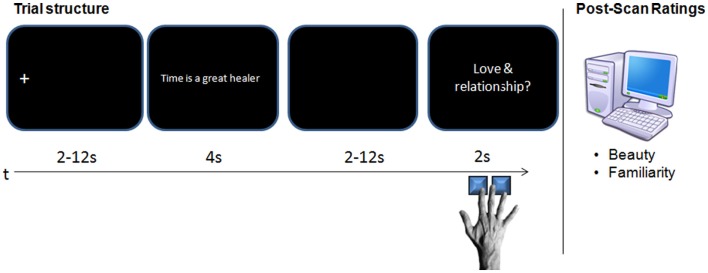
**Timeline of one trial in the fMRI-scanner**.

After task practise and instruction outside of the scanner, participants underwent a scanning session consisting of five imaging runs with 40 trials each. Each trial started with a fixation cross at the position of the first letter, followed by a sentence presented in one line of white letters on a black background (Arial; 18 pt; left aligned; 4 s), and a blank screen after which a verbal category cue (*everyday life*, *health and well-being*, *love and relationship*, or *work and success*) was presented on the screen (2 s). The blank screen and fixation cross were jittered (2–12 s; mean 5 s, respectively). Participants were instructed to read silently, and to indicate whether or not an item fitted into the provided semantic category by pressing a button within the response window of 2 s, during which the category cue was shown. Four independent raters had rated the fit of each item into each category before, so that across participants, each item was paired equally often with a fitting and a non-fitting category (defined as categories on which all four raters agreed). Within participants, the number of fitting and non-fitting category cues was also balanced. Each run comprised eight items per condition. The order of items in each condition was counterbalanced across participants/runs using a Latin Square design to rule out sequence-effects. Stimuli were pseudo-randomly mixed within each run. Timing and order of stimulus presentation were optimized for estimation efficiency using rsfgen (AFNI)^2^. Presentation (Neurobs, Inc., Albany, CA, USA)^3^ was used for stimulus delivery and timing on a Dell computer under Windows XP. Visual stimuli were presented using MR-compatible LCD goggles (Resonance Technology Inc., Northridge, CA, USA), and the computer was synchronized with the onset of each functional run to ensure the accuracy of event timing. Participants responded with their right hand via a MR-compatible button box. Following the functional scan, participants gave explicit esthetic judgments outside of the MR by rating each item on overall “beauty” (allowing for consideration of stylistic quality, pleasantness, but also approval of the social or moral value implied in a proverb) on a seven-point Likert scale ranging from 1 (*not beautiful at all*) to 7 (*very beautiful*). Afterward, the participants were asked if they had encountered the item in exactly this wording ever before, prior to the experiment. For this task, they made use of a seven-point confidence scale ranging from −3 (*definitely unfamiliar*) to +3 (*definitely familiar*). Values around zero indicate that they were unsure whether they had encountered the expression before. The familiarity rating served as a measure to control if the participants had really known the “familiar” items before and if the “unfamiliar proverbs,” proverb-substitutions and proverb-variants had been novel to them. For an illustration of the task and procedure see Figure 1.

### fMRI acquisition

Imaging was performed using a 3T Siemens (Erlangen, Germany) Tim Trio MRI scanner fitted with a 12-channel head coil at the Dahlem Institute for Neuroimaging of Emotion (DINE). In each of five imaging runs, 320 whole brain functional T2*-weighted echoplanar images (EPI) [slice thickness, 3 mm; no gap; 37 slices; repetition time (TR), 2 s; echo time (TE), 30 ms; flip angle, 90°; matrix, 64 × 64; field of view (FOV), 192 mm; voxel-size 3.0 mm × 3.0 mm × 3.0 mm] were acquired. Four additional volumes were discarded at the beginning of each run to allow for T1 equilibrium effects. Parallel image reconstruction with GRAPPA was enabled, acceleration factor of 2 was engaged. Additionally, a T1-weighted matched-bandwidth high-resolution (voxel-size 1.0 mm × 1.0 mm × 1.0 mm) anatomical scan (same slice prescription as EPI), and magnetization-prepared rapid-acquisition gradient echo (MPRAGE) were acquired for each participant for registration (TR, 1.9; TE, 2.52; FOV, 256; matrix, 256 × 256; sagittal plane; slice thickness, 1 mm; 176 slices).

### Data analysis

Behavioral data from the semantic categorization task and from the post-scan ratings were analyzed using repeated measures analysis of variance (ANOVA) in PASW 18 (IBM SPSS Statistics). The only interest in the semantic categorization data was to check if participants performed above chance level, which was interpreted as indicating that participants had achieved at least some access to the semantic meaning of the items. The main purpose of the semantic categorization task was to keep participants’ attendance high throughout the experiment. Interpreting any “accuracies” or reaction time data seemed unreliable. Proverbs can have manifold interpretations and the item difficulty in the semantic categorization task could not be controlled, so even a “wrong” answer would have been no hard proof for misunderstanding (because the participant might have had a different, but equally valid interpretation in mind). Therefore, “accuracies” and reaction times were not modeled in the analysis of the functional data. This was, however, unproblematic, because the regressors that modeled the conditions covered the 4 s period during stimulus presentation prior to the semantic categorization task. BrainVoyager QX 2.0 (Brain Innovation, Maastricht, The Netherlands) was used to analyze the recorded MRI data (Goebel et al., 2006). The functional data were slice-scan time corrected (cubic-spline interpolation) to correct for the sequentially executed interleaved slice acquisition and motion corrected. Intra-session image alignment to correct for motion across runs was performed using the first image of the first functional run as the reference image. Following linear trend removal, data was filtered temporally in 3D with a high pass Fourier filter of two cycles in time course to remove low frequency drifts. Preprocessed data were spatially smoothed using an 8 mm full-width-half-maximum Gaussian kernel to reduce noise. For spatial normalization the individual T1 images were transformed into Talairach space (Talairach and Tournoux, 1988) and all statistical analyses were performed in Talairach space. Anatomical regions were identified by manual inspection using the Talairach atlas and the Talairach demon^4^.

The statistical analyses were carried out using a voxel-wise General Linear Model (GLM) at the single-participant-level first, based on design matrices, which included the estimated 3D motion parameters obtained during preprocessing as well as predictors for all task conditions and the button-response window. Separate regressors per condition were modeled using a boxcar function with the length of the duration of the stimulus presentation (4 s per trial), which was convolved with a theoretical Two Gamma hemodynamic response function (Friston et al., 1998), and the model was independently fitted to the signal of each voxel. Fixation periods were not modeled and the response period during the semantic categorization task was modeled as a regressor of no interest. These estimates of the trial responses relative to baseline were subsequently combined to provide an estimate of the condition effects, which could then be used to contrast the experimental conditions. The reported group analyses were conducted following a random effects model. Analysis space was covering the whole brain, head and skull tissue excluded. Unless stated otherwise, the correction level for reported activations was *p* < 0.05 [based on an initial voxel-level threshold of *p* (uncorrected) < 0.005]. To control for Type I error, the uncorrected maps were entered into a Monte Carlo simulation to determine the cluster size correction level. Clusters below the correction level are neither reported nor visualized. To determine familiarity effects, familiar, and unfamiliar proverbs were directly contrasted against each other, as well as contrasted separately against non-rhetorical sentences. To discover brain regions sensitive to both types of defamiliarization, a conjunction analysis [(proverb-substitutions > familiar proverbs) and (proverb-variants > familiar proverbs)] was carried out. To specify which brain regions were sensitive to the type of defamiliarization, proverb-variants were directly contrasted with proverb-substitutions.

## Results

### Behavioral results

Task performance during the distracter task was analyzed to check participants’ involvement during the fMRI experiment. The mean accuracy of the semantic categorization task during the experiment was significantly above the 50% chance level for all conditions (see Table 4), rendering it highly likely that participants were engaged in interpreting the semantic meaning of the items in all conditions. The mean response times in the semantic categorization task were comparable between sentence types, *F*(4,22) = 0.93, *p* > 0.05, indicating equal task difficulty across conditions. Post-scan familiarity ratings were analyzed to validate stimulus categorization into “familiar” and “unfamiliar” conditions. Significantly higher familiarity ratings were assigned to “familiar” proverbs than to all other conditions (all *p*-values < 0.001), thereby validating our categorization. Familiarity ratings of two participants were lost due to a programming error. To test the three theories described in the introduction, the ratings on the beauty scale were analyzed. Significant differences in beauty ratings between types of sentence were found [*F*(2.8,71) = 21.46, *p* < 0.001, ${\text{η}}_{p}^{2}=0.46$]. *Post hoc* comparisons revealed that in line with the hedonic fluency hypothesis, familiar proverbs were judged as significantly more beautiful than all other conditions (all *p*-values < 0.001). However, the finding that proverb-substitutions were significantly less beautiful than unfamiliar proverbs (*p* < 0.01) and familiar proverbs (*p* < 0.001), but comparable to proverb-variants and non-rhetorical sentences was not predicted by any of the theories; nor was the finding that unfamiliar proverbs, proverb-variants, and non-rhetorical sentences received equal beauty ratings. These results will be discussed later. Table 4 lists the Means and SDs of all behavioral measures.

**Table 4:** **Behavioral results**.

	Semantic categorization task				Post-Scan Rating			
	Accuracy^1^		RT (ms)		Familiarity^2^		Beauty^3^	
	*M*	SD	*M*	SD	*M*	*SD*	*M*	*SD*
Familiar	77.50	11.98	2142	340	2.32	0.40	4.84	0.81
Unfamiliar	74.71	11.45	2188	353	−1.69	1.01	4.03	0.92
Variantes	69.13	11.51	2142	327	−1.93	0.98	3.69	1.06
Substitutions	77.79	10.66	2184	314	−1.52	1.07	3.35	0.82
Non-rhetor	72.40	12.30	2087	362	−0.57	1.24	3.66	0.91
Significances	*F*(4,100) = 8.46, *p* < 0.001, ${\text{η}}_{p}^{2}=0.25$		n.s.		*F*(3,68.7) = 144.67, *p* < 0.001, ${\text{η}}_{p}^{2}=0.86$		*F*(11.9,0.5) = 21.46, *p* < 0.001, ${\text{η}}_{p}^{2}=0.46$	

*^1^Proverb-variants had significantly lower accuracy levels than familiar, unfamiliar, and proverb-substitutions (all *p* < 0.05)*.

*^2^Familiar proverbs were significantly more familiar than all other conditions (all *p* < 0.001). Non-rhetorical sentences were significantly less familiar than familiar proverbs, but more familiar than unfamiliar, violated, and proverb-variants (all *p* < 0.001)*.

*^3^Familiar proverbs had significantly higher beauty ratings than all other conditions (all *p* < 0.001). Proverb-substitutions had significantly lower beauty ratings than unfamiliar proverbs (*p* < 0.01)*.

### Imaging results

#### Effect of familiarity

To investigate how familiarity affects the reading process, we contrasted familiar proverbs against unfamiliar proverbs. The hedonic fluency hypothesis would have predicted a stronger contribution of affect-related regions on reading familiar proverbs, while foregrounding theory would have predicted an activation of affect-related regions for unfamiliar proverbs.

**Figure 2:**
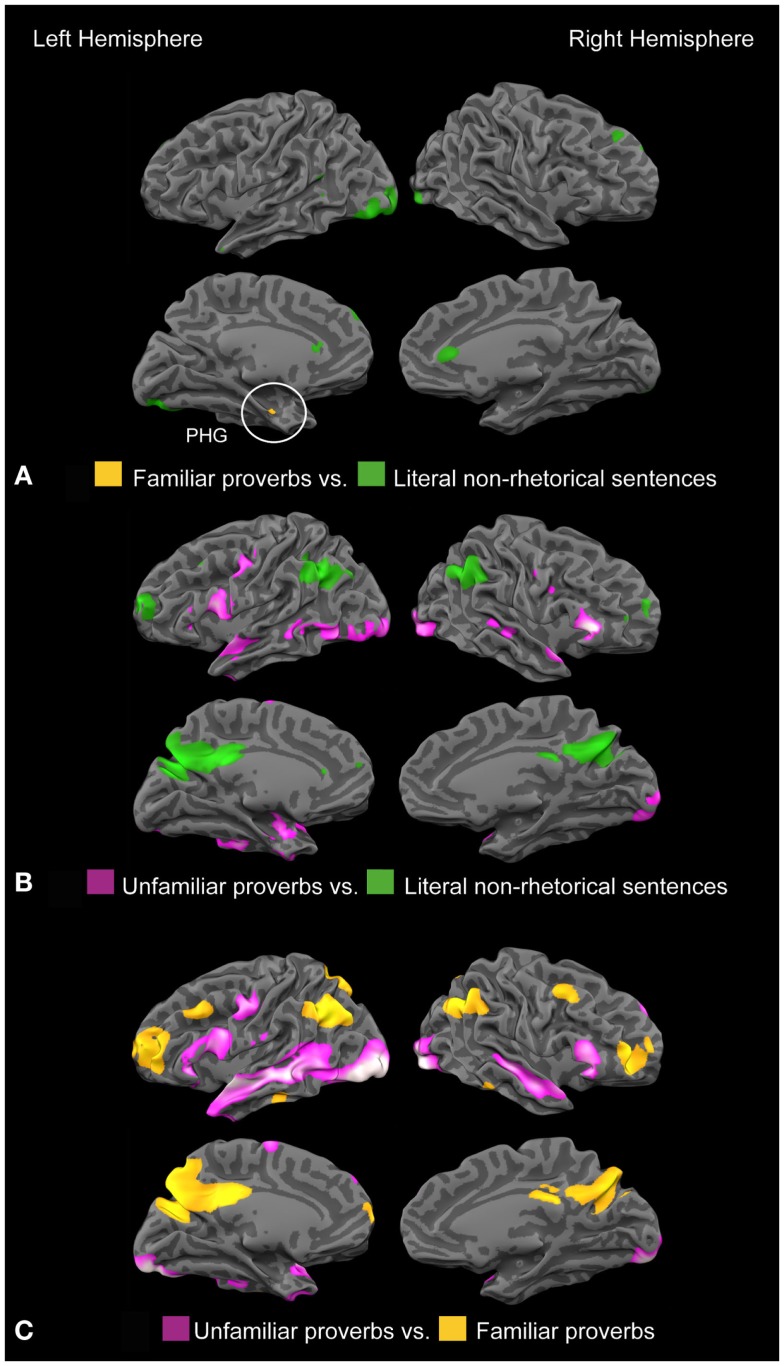
**(A)** Contrast familiar proverbs – non-rhetorical sentences activate the left parahippocampal gyrus (*X* = −33, *Y* = −10, *Z* = −17; *z* = 3.92). **(B)** Contrast unfamiliar proverbs – non-rhetorical sentences activates a bilateral frontotemporal network. **(C)** Contrast unfamiliar – familiar proverbs: familiar proverbs elicited a bilateral activation pattern similar to the dorsomedial part of the default network. Unfamiliar proverbs activated areas related to sentence reading, comprising biltaeral MTG/STG (BA 21, 22, 38), bilateral occipital cortex (BA 18/19), posterior rostral part of the medial prefrontal cortex (prMFC, BA 9) and bilateral parts of the motor and premotor cortex (BA 4/6).

In the direct contrast “familiar-unfamiliar” shown in Figure 2C, familiar proverbs elicited a bilateral activation pattern similar to the dorsomedial part of the default network (Buckner et al., 2008). In the reversed contrast, unfamiliar proverbs showed relatively stronger activity in areas related to sentence reading, comprising nearly the whole left superior and middle temporal lobe, as well as the right MTG/STG (BA 21, 22, 38), bilateral occipital cortex (BA 18/19), and cerebellum. Additionally, the bilateral posterior rostral part of the medial prefrontal cortex (prMFC, BA 9) and bilateral parts of the motor and premotor cortex (BA 4/6) were activated. A list of all coordinates and statistical values is provided in the Table A1 in Appendix. The activation pattern for familiar proverbs was similar to the default mode network, indicating that familiar proverbs were easier to process than unfamiliar proverbs. No differences in activation were found in reward-related regions (OFC, nucleus accumbens, ventral striatum, ACC). The activation pattern for unfamiliar proverbs included several areas associated with affective processing, such as the temporal poles and the medial prefrontal cortex; however, these regions are also sensitive to cognitive load.

When familiar and unfamiliar proverbs were separately contrasted against non-rhetorical, literal sentences, familiar proverbs recruited the anterior part of the left parahippocampal gyrus (PHG), whereas unfamiliar proverbs activated a broad bilateral frontotemporal network in the semantic system (Binder et al., 2009). These findings are illustrated in Figures 2A,B. None of the classical reading-related frontotemporal areas emerged from the contrast “familiar/non-rhetorical,” suggesting that the two conditions relied on them in equal measure. In line with recent findings on figurative language processing, we found RH involvement only for novel, but not for familiar proverbs (Faust and Mashal, 2007; Schmidt et al., 2007; Pobric et al., 2008; Yang et al., 2009; Cardillo et al., 2012). Affect-related brain regions were predominantly observed as related to unfamiliar proverbs, being in line with foregrounding theory and the concept of defamiliarization rather than with the hedonic fluency hypothesis.

#### Effect of defamiliarization

**Figure 3:**
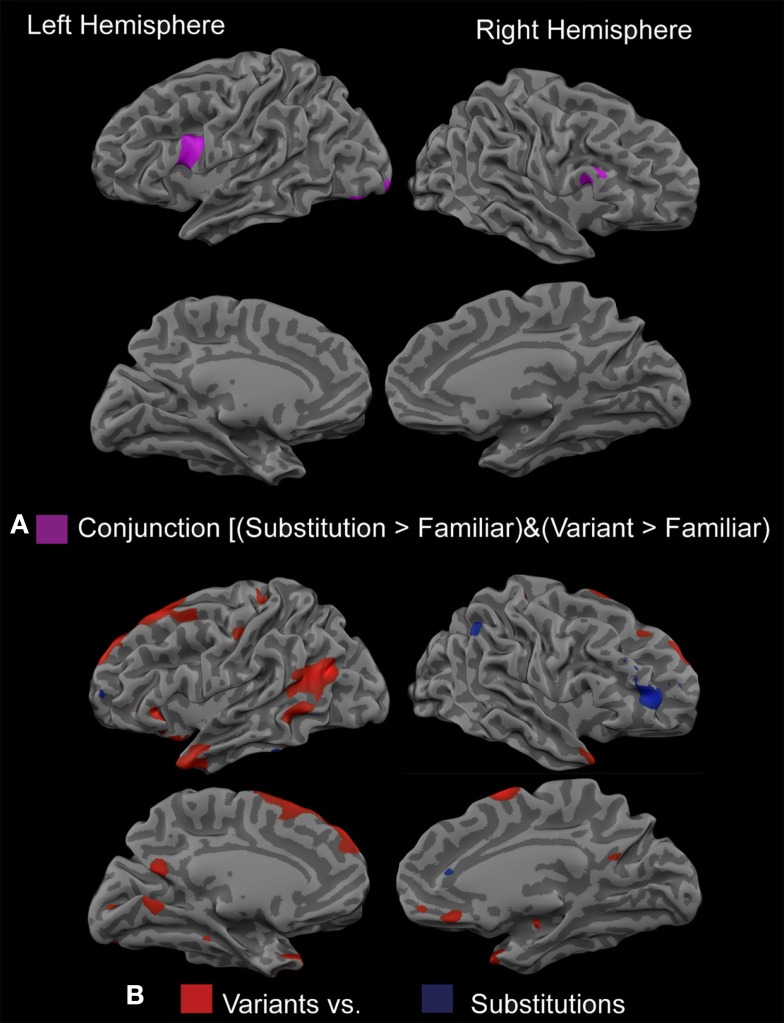
**Effect of defamiliarization. (A)** Conjunction analysis [(proverb-substitutions > familiar proverbs) and (proverb-variants > familiar proverbs) activated bilateral IFG (LH: BA44; RH: BA9) and bilateral inferior occipital gyrus (IOG; BA18/19). **(B)** Contrast proverb-variants – proverb-substitutions: Proverb-variants recruited bilateral temporal poles (BA 38), medial prefrontal area (subcallosal ACC, vmPFC, dmPFC), posterior cingulate region (PCC/cuneus), parahippocampal gyri, bilateral occipital cortex, bilateral IFG (BA 47), and left MTG/STG. Proverb-substitutions activated the dorsal part of the ACC, right dlPFC (BA 10), left vlPFC, right IPL (BA40), and left fusiform gyrus.

To investigate general effects of defamiliarization across types of defamiliarization, a conjunction analysis [(proverb-substitutions > familiar proverbs) and (proverb-variants > familiar proverbs)] was calculated. It revealed that the bilateral IFG (LH: BA44; RH: BA9) and bilateral inferior occipital gyrus (IOG; BA18/19) were activated significantly stronger for both versions of defamiliarized proverbs (proverb-variants and proverb-substitutions) than for familiar proverbs (Figure 3A). The lack of activation in affect-related areas hints toward a more cognitive effect of defamiliarization itself. However, both foregrounding theory and the optimal innovation hypothesis would imply a stronger affective involvement for innovative proverb-variants than for proverb-substitutions. To uncover regions sensitive to the type of defamiliarization, proverb-variants were directly contrasted with proverb-substitutions (Table A2 in appendix). Proverb-variants activated areas related to affective evaluation, such as the bilateral temporal poles (BA 38), medial prefrontal area (medial OFC, vmPFC, dmPFC), and posterior cingulate region (PCC/cuneus), as well as the parahippocampal gyri. Furthermore, regions probably associated with visual attention, such as the bilateral occipital cortex, and regions relevant for semantic integration and sentence processing (bilateral IFG, BA 47 and left MTG/STG) were activated more strongly by proverb-variants than by proverb-substitutions. Proverb-substitutions, however, recruited areas mostly related to cognitive evaluation and error detection, such as a cluster in the dorsal ACC, right dlPFC (BA 10), left anterior frontal cortex, right IPL (BA40), and left fusiform gyrus. In summary, while proverb-substitutions were associated with activation of the frontoparietal attention network, proverb-variants engaged the affect-related medial prefrontal and medial temporal regions (Figure 3B), which can be explained by foregrounding theory and the optimal innovation hypothesis, but not by hedonic fluency.

## Discussion

This study analyzed explicit reader response and neural correlates related to the reading of proverbs, in order to investigate the contribution of familiarity and defamiliarization on the affective and esthetic perception of literature, exemplified by proverbs. Familiarity and the degree of defamiliarization were manipulated. We conclude that familiarity and defamiliarization are two distinct components that can influence the affective and esthetic processing of literature. After discussing the neural correlates of familiarity and defamiliarization separately, we will turn to a general discussion of their implications for an esthetic perception of literature.

### The familiarity effect

The rating data indicate that familiarity can affect the esthetic perception of sentences: Familiar proverbs received significantly higher beauty ratings than all other conditions. However, the hedonic fluency hypothesis would have predicted a linear correlation between beauty ratings and complexity, which was not found. Instead, relatively simple literal sentences were not rated significantly different from cognitively challenging unfamiliar proverbs, the latter even ranked second in beauty ratings. These non-linear results suggest that other parameters beside fluency influenced the explicit esthetic evaluation. The fMRI data show that in addition to the increased demands of cognitive processing, more affective processing (in the amygdala, temporal poles, and medPFC) is triggered by the unfamiliar proverbs. Although affective processing *per se* does not predict a positive or negative esthetic judgment, it probably still affects the evaluation process; in the current experiment, it resulted in a relatively high evaluation of beauty, even though the conditions were matched in terms of valence and arousal.

In summary, although familiar proverbs were the condition with the highest processing fluency, the result that they were singled out by beauty ratings is probably not only based on processing fluency. While their rhetorical features may account for their cultural success and familiarity in the first place, their success in the beauty ratings may be based on the successful recognition of familiar items in the context of novel and defamiliarized ones.

### General effect of defamiliarization

In the present experiment, two conditions (proverb-substitutions and proverb-variants) represented defamiliarized versions of familiar German proverbs. Only the proverb-variants fulfilled the criterion of being “optimally innovative” as defined by Giora et al. (2004). Common to both conditions, the bilateral IFG and left IOG responded more strongly to the defamiliarized version than to the original proverb. The LIFG is one of the most frequently found regions in neuroimaging studies on semantics and language processing in general. We had expected to find the more semantic ventral part, the pars orbitalis and triangularis, to be associated with defamiliarization. Surprisingly, we found activation of the more syntactic dorsal part of the pars opercularis (BA 44) instead (Dapretto and Bookheimer, 1999; Friederici et al., 2000; Newman et al., 2010). This encourages the interpretation that both proverb-variants and proverb-substitutions share an enhanced demand for syntactic processing relative to the formulaic structure of the familiar proverb. If defamiliarization destroys the expected structure of the familiar proverb, further syntactic processing becomes necessary. However, semantic processing is not ruled out, as both the ventral and dorsal LIFG have been shown to be related to semantic memory in a recent lesion study (Yang et al., 2010). In the present experiment, we did find stronger activation of the more semantic ventral part of the LIFG by proverb-variants than by proverb-substitutions. We attribute the activation of the RIFG and the enhanced activation of the visual areas to attention shifts toward the unexpected word, as the RIFG has recently been found to be involved in the detection of relevant, unexpected stimuli (Corbetta and Shulman, 2002; Hampshire et al., 2010). Interestingly, neighboring areas in the prefrontal cortex and the occipital gyri have also been found in experiments on art perception, where they have been associated with viewing pictures in an esthetic rather than a pragmatic mode (Cupchik et al., 2009). This observation is in line with the claim of foregrounding theory that defamiliarization leads to esthetic perception.

In summary, our results indicate that the technique of defamiliarization effectively draws attention to stimuli that would not have been further considered in their conventional form. We interpret this internal attention shift as a sign of participants entering an esthetic mode of perception. However, contrary to what a strict interpretation of foregrounding theory would predict, defamiliarization as such does not elicit spontaneous affective evaluation, nor are defamiliarized items generally judged as especially beautiful.

### Optimal innovation

The key prediction of the optimal innovation hypothesis says that optimally innovative proverb-variants should be processed with stronger affective involvement and therefore be preferred over all other conditions. In the present experiment, only the proverb-variants fulfilled all criteria of “optimal innovativeness” (Giora et al., 2004), whereas the proverb-substitutions had the same semantic content as the corresponding familiar proverbs. The participants noticed this difference and rated the inventiveness of the proverb-substitutions significantly lower. The rating data thus do not confirm the optimal innovation hypothesis, because proverb-variants were considered neither more beautiful nor more inventive than familiar and unfamiliar proverbs. However, the fMRI data revealed processing differences between the two types of defamiliarized proverbs. Consistent with the optimal innovation hypothesis, the proverb-variants activated brain regions associated with self-related emotional memories, comprising the bilateral anterior temporal lobes, dmPFC, medial OFC, cuneus, and bilateral PHC including the right amygdala. We interpret the relatively greater involvement of these regions as enhanced affective processing elicited by the proverb-variants. Reading proverb-variants also required a greater semantic integration effort because proverb-variants evoke two contrasting responses (that of the familiar proverb and the novel word) that have to be related. This integration effort is related to the stronger ventral IFG activation for proverb-variants than for proverb-substitutions. The medial OFC frequently associated with monitoring, learning, and memory of reward value (see Kringelbach and Rolls, 2004, for a review) could reflect the rewarding aspect of successful semantic integration. Comparable frontomedian activation was found for self-referential processing (Zysset et al., 2002), explicit esthetic judgments (Jacobsen et al., 2006), and idiom comprehension (Lauro et al., 2008). Lauro and colleagues showed that activation in the frontomedian area increased the functional connection between bilateral frontotemporal areas during idiomatic processing, thus assigning the frontomedian area a key role in the selection between alternative sentence meanings. Proverb-variants most certainly triggered a similar comparison between the content of the familiar proverb that echoes in the background and the content of the proverb-variant which cognitively comes to the foreground (Jacobs, 2011).

One might wonder why in spite of more intense affective processing the resulting judgment is not one of high “beauty” (a term widely associated with something good and pleasurable). An explanation can be found in the specific characteristics of proverbs: proverbs are used to convey moral and social values, which are questioned by the alternative content of proverb-variants. The affect-related areas that we found have also been attributed to moral emotions (Heekeren et al., 2003). While proverbs express traditionally valued and accepted cultural norms and beliefs, proverb-variants often oppose, or at least question the traditional value. Proverb-substitutions, which do not question the content of the corresponding familiar proverbs, did not recruit this moral emotion network; instead, they activate the right IPL, left fusiform gyrus, and the ACC which are associated with attention shifting, error detection, and conflict management. In short, although both types of defamiliarization seem to enhance attention, only proverb-variants that included a conceptual mismatch were correlated with affective/moral evaluation. Proverb-substitutions that only provided formal defamiliarization received very low beauty ratings. Functional data suggest that this less innovative condition may have been processed as containing errors. Importantly, enhanced affective evaluation does not necessarily result in a positive esthetic judgment.

### General discussion

In the present fMRI experiment we tested three hypotheses of how cognitive processing is linked to esthetic perception. Concerning the esthetic perception of literature, our data yield mixed results. We agree with Giora that neither high processing fluency nor novelty is a sufficient precondition to elicit pleasure. However, the predictions of the optimal innovation hypothesis were not met, as proverb-variants did not stand out from the other categories in terms of beauty. This might be due to “non-optimal” stimuli, but for the optimal innovation hypothesis to make valid predictions, one would need a more elaborated concept of how to “optimally” combine familiar and novel elements, which will be difficult to define, and many more dimensions apart from familiarity, such as aptness, or imageability might have to be considered when estimating preference for figurative expressions, as these dimensions have been shown to be important in metaphor processing (Gerrig and Healy, 1983; Marschark et al., 1983; Katz et al., 1988; McGlone, 2007). Our rating data support the hedonic fluency theory, while the functional data are consistent with the foregrounding theory. Beauty ratings singled out familiar proverbs thus supporting the hedonic fluency theory and preference for prototype models (Martindale and Moore, 1988; Martindale et al., 1990). The behavioral results demonstrate the hedonic value of familiarity, represented on a neural level by the activation of the left PHC by familiar proverbs rather than by baseline sentences. The resulting feeling of familiarity might be considered a safety signal and carry hedonic value. However, the hedonic fluency model alone cannot account for the whole behavioral pattern, e.g., for the fact that unfamiliar proverbs (the condition with the lowest familiarity and a high processing effort) ranked second in “beauty.” According to foregrounding theory, unfamiliar proverbs confront the reader with a condensed content that is enhanced by stylistic devices. Items high in foregrounding and defamiliarization were expected to set the reader into a mode of esthetic perception. If used in a text, they stand out against the background of fluent and literal language and offer the closing of a new meaning gestalt. During the process of reading, the readers might switch from an automatic reading of “background” information to a slower, cognitively more demanding reading mode whenever they encounter “foregrounded” passages (Iser, 1974; Jacobs, 2011). Functional neuroimaging data are consistent with this aspect of foregrounding theory. Relative to unfamiliar proverbs, familiar proverbs, and non-rhetorical baseline sentences engaged the default mode network which is associated with mentalizing, imagination, and self-referential thinking, probably due to the lower cognitive demand. These relatively easily processed conditions may have served as a “background” for the more difficult-to-process, unfamiliar proverbs. Proverb-variants recruited a network of self-referential moral evaluation, suggesting that a certain amount of conceptual defamiliarization (violating world-knowledge or moral standards) might effectively trigger affective evaluation. However, unlike previous behavioral studies (van Peer, 1986; Miall and Kuiken, 1994; Hakemulder, 2004), we did not observe a positive relation of foregrounding and explicit esthetic judgment. The reason for this might be exactly that proverbs characteristically imply a social or moral value. Proverb-variants naturally question these traditional values, and account for some interindividual variation on the esthetic value of such a critical statement. Nevertheless, we propose that in literature and poetry, passages that question world-knowledge or moral values have a high foregrounding potential as they might automatically trigger affective evaluations that feed into the esthetic judgment.

However, the finding that the most fluent, prototypical stimuli were preferred over less fluent conditions should not be generalized too rashly. As the results of Menenti et al. (2009) suggest, the perception of a sentence or a phrase is strongly influenced by the context in which it appears: whether a fluent text is appreciated for its readability or rejected because of its low quality can be strongly genre-dependent (Galak and Nelson, 2011). More generally, the semantic context of an artwork is known to modulate esthetic judgments (Kirk et al., 2009), especially if it is embedded in a narrative or a pictorial composition. Hence, future studies should try to shed light on the interaction of text quality, content, and context. Furthermore, expert and non-expert readers, whose ways of reaching esthetic judgments might differ (Hekkert and van Wieringen, 1996), represent just one example of interindividual differences that would be worth addressing in further studies. Our findings emphasize that in the case of complex linguistic structures such as sentences, figurative language, and ultimately text, no single factor is likely to explain all of the dimensions of an esthetic judgment. To specify the role of figurative language and stylistic devices in the esthetic perception of literature, an elaborated foregrounding theory (ideally fed by classic rhetorical theory) allowing for more specific predictions about the esthetic and affective potential of rhetorical language may be necessary.

## Conflict of Interest Statement

The authors declare that the research was conducted in the absence of any commercial or financial relationships that could be construed as a potential conflict of interest.

## Footnotes

^1^http://wortschatz.uni-leipzig.de

^2^http://afni.nimh.nih.gov

^3^www.neurobs.com

^4^http://www.talairach.org

## Appendix

### List of stimuli

#### Experimental conditions

**Table:**

Familiar proverbs	Proverb-substitutions	Proverb-variants
**Wissen** ist Macht	**Kenntni** *s* ist Macht	**Gewissen** ist Macht
Wer **wagt**, gewinnt	Wer **riskiert**, gewinnt	Wer **fragt**, gewinnt
Reden ist Silber, **Schweigen** ist Gold	Reden ist Silber, **Stille** ist Gold	Reden ist Silber, **Helfen** ist Gold
Je später der Abend, desto schöner die **Gäste**	Je später der Abend, desto schöner die **Besucher**	Je später der Abend, desto schöner die **Gesten**
**Ende** gut alles gut	**Schluss** gut, alles gut	**Rente** gut, alles gut
Gut Ding will **Weile** haben	Gut Ding will **Zeit** haben	Gut Ding will **Freude** haben
Die Zeit heilt alle **Wunden**	Die Zeit heilt alle **Schmerzen**	Die Zeit heilt alle **Wunder**
Übung macht den **Meister**	Übung macht den **Könner**	Übung macht den **Muskel**
Eine Hand **wäscht** die andere	Eine Hand **reinigt** die andere	Eine Hand **wärmt** die andere
In der **Ruhe** liegt die Kraft	In der **Stille** liegt die Kraft	In der **Natur** liegt die Kraft
Jeder **Topf** hat einen Deckel	Jeder **Pott** hat einen Deckel	Jeder **Sarg** hat einen Deckel
Auch Rom ist nicht an einem Tag **erbaut** worden	Auch Rom ist nicht an einem Tag **erstellt** worden	Auch Rom ist nicht an einem Tag **zerstört** worden
Ein gesunder **Geist** wohnt in einem gesunden Körper	Ein gesunder **Sinn** wohnt in einem gesunden Körper	Ein gesunder **Schnaps** wohnt in einem gesunden Körper
Andere Zeiten, andere **Sitten**	Andere Zeiten, andere **Bräuche**	Andere Zeiten, andere **Männer**
Alle **Wege** führen nach Rom	Alle **Pfade** führen nach Rom	Alle **Sünden** führen nach Rom
Geld allein macht nicht **glücklich**	Geld allein macht nicht **fröhlich**	Geld allein macht nicht **ehrlich**
Früh übt sich, wer ein **Meister** werden will	Früh übt sich, wer ein **Könner** werden will	Früh übt sich, wer ein **Rentner** werden will
Vorsicht ist die Mutter der **Porzellankiste**	Vorsicht ist die Mutter der **Glasvitrine**	Vorsicht ist die Mutter der **Karriere**
Sauer macht **lustig**	Sauer macht **freudig**	Sauer macht **durstig**
Handwerk hat **goldenen** Boden	Handwerk hat **edlen** Boden	Handwerk hat **harten** Boden
Ein **Unglück** kommt selten allein	Ein **Schaden** kommt selten allein	Ein **Zwilling** kommt selten allein
Keine Rose ohne **Dornen**	Keine Rose ohne **Spitzen**	Keine Rose ohne **Duft**
Müßiggang ist aller **Laster** Anfang	Müßiggang ist aller **Fehler** Anfang	Müßiggang ist aller **Freude** Anfang
Wer Wind säht, wird **Sturm** ernten	Wer Wind säht wird **Orkan** ernten	Wer Wind säht, wird **Energie** ernten
Wer nicht hören will muss **fühlen**	Wer nicht hören will muss **spüren**	Wer nicht hören will, muss **lesen**
Wer schön sein will muss **leiden**	Wer schön sein will muss **erdulden**	Wer schön sein will muss **genießen**
Viele Köche **verderben** den Brei	Viele Köche **ruinieren** den Brei	Viele Köche **kreieren** den Brei
Wer hoch steigt, kann tief **fallen**	Wer hoch steigt, kann tief **stürzen**	Wer hoch steigt, kann tief **schauen**
**Ordnung** ist das halbe Leben	**System** ist das halbe Leben	**Neugier** ist das halbe Leben
Gelegenheit macht **Diebe**	Gelegenheit macht **Räuber**	Gelegenheit macht **Liebe**
Der Fisch fängt vom Kopf her an zu **stinken**	Der Fisch fängt vom Kopf her an zu **muffeln**	Der Fisch fängt vom Kopf her an zu **trinken**
**Geld** regiert die Welt	**Gold** regiert die Welt	**Neid** regiert die Welt
**Liebe** macht blind	**Verliebtheit** macht blind	**Werbung** macht blind
Was der Bauer nicht kennt, **frisst** er nicht	Was der Bauer nicht kennt, **isst** er nicht	Was der Bauer nicht kennt, **melkt** er nicht
Zeit **ist** Geld	Zeit **bedeutet** Geld	Zeit **frisst** Geld
**Alter** hilft vor Torheit nicht	**Bejahrtheit** hilft vor Torheit nicht	**Bildung** hilft vor Torheit nicht
Sport ist **Mord**	Sport ist **Tötung**	Sport ist **Mode**
Rache ist **süß**	Rache ist **süßlich**	Rache ist **fies**
Der Mensch denkt, **Gott** lenkt	Der Mensch denkt, **Herrgott** lenkt	Der Mensch denkt, **Computer** lenkt
Kleider machen **Leute**	Kleider machen **Menschen**	Kleider machen **Neider**

#### Control conditions

**Table:**

Non-rhetorical sentences	Unfamiliar proverbs
Etwas Sport ist gesund	Jahre lehren mehr als Bücher
Lachen entspannt im Alltag	Gutes Essen lässt Sorgen vergessen
Kerzen machen einen Raum heller	Heiter kommt weiter
Offene Menschen haben viele Freunde	Andrer Mann, andres Glück
Wissen ist im Beruf nützlich	Einfalt hat schöne Gestalt
Ein Hausbau braucht viel Zeit	Kleiner Mann, großes Herz
Jeder Schmerz endet irgendwann	Nicht alle Wolken regnen
Ruhige Leute ertragen Stress gut	Kluge Leute fehlen auch
Was man gut kann, macht Spaß	Adler fangen keine Mücken
Fleiß führt oft zum Erfolg	Natur geht vor Lehre
Fast jeder findet mal einen Partner	Was der Löwe nicht kann, das kann der Fuchs
Man soll das Leben heiter verbringen	Die Wahrheit ist zu schlau um gefangen zu werden
Mit Training stärkt sich der Körper	Wer will, was er kann, fängt nichts vergeblich an
Ein Witz lockert die Stimmung	Neuer Freund, neuer Wein
Salz hebt den Geschmack	Das Herz lügt nicht
Ein Reicher hat im Leben kaum Sorgen	Das Glück muss man erobern
Kinder sollte man jung fördern	Was nicht rastet und nicht ruht, tut in die Länge nicht gut
Man sollte nicht lang zornig sein	Behaupten ist nicht beweisen
Den Müll zu trennen ist klug	Der Edle zürnt nicht lange
Gemüse ist so gesund wie Obst	Besser umkehren als irregehn
An Feiertagen gibt es oft Stau	Karges Weib geht oft zur Kiste
Auch das Schöne hat Nachteile	Kalt Eisen brennt nicht
Brüder kämpfen oft um das Erbe	Besser karg als arg
Pflanzen, die man nicht gießt, welken	Besser beneidet als beklagt
Viele Beziehungen halten nicht	Nesseln brennen Freund und Feinde
Für Schönheit wird zu viel Geld vergeudet	Das Böse glaubt man gern
Jemand schwaches ist eher hilflos	Ein einzelner Armreif klappert nicht
Ohne Geld gehen Firmen pleite	Das Alte klappert, das Neue klingt
Mit Hektik richtet man nur Schaden an	Pfaffen segnen sich zuerst
Es ist schwer Fehler zu gestehen	Bosheit ist bald gelernt
Schlechte Anführer schaden der Gruppe	Besser die Hand in einem Kuhfladen denn in fremdem Gelde
Naive Leute werden ausgenutzt	Bruderzwist gar heftig ist
Ein Gerücht wird rasch weiter erzählt	Den Esel will jedermann reiten
Im Winter muss man viel anziehen	Jeden Morgen neue Sorgen
Man sollte Zeit nicht vergeuden	Amt ohne Sold macht Diebe
Zu laute Musik schadet den Ohren	Kein Kranz schützt vor Kopfweh
Wer feig ist hat oft Angst	Geschieht’s, man sieht’s
Einsamkeit macht viele Leute krank	Borgen tut nur einmal wohl
Sorgen drücken auf die Laune	Viel Wissen, wenig Gewissen
Alkohol macht eher gereizt	Ein fauler Apfel macht zehn

*Note. Critical words that differ between familiar proverbs, proverb-substitutions, and proverb-variants are written in bold*.

### Activation lists

**Table A1:** **Effects of familiarity**.

Region	Sub-region		Activation maximum			Volume (mm^3^)	Maximum *Z*-score
			*x*	*y*	*z*
**FAMILIAR PROVERBS > UNFAMILIAR PROVERBS**							
L	Middle frontal gyrus (BA 10)		−36	50	13	10665	5.31
	Superior frontal gyrus (BA 10)		−24	62	16	81	5.01
	Medial frontal gyrus (BA 10)		−12	65	4	81	4.00
L	Middle frontal gyrus (BA 8)		−39	23	40	2538	3.80
L	Inferior temporal gyrus (BA 20)		−57	−28	−17	621	3.77
L	Inferior parietal lobule (BA 40)		−36	−55	37	10017	5.25
	Precuneus (BA 19)		−36	−73	34	513	5.15
L	Cuneus (BA 7)		−9	−67	28	26190	5.83
	Posterior cingulate (BA 23)		−3	−34	25	6291	5.39
	Precuneus (BA 7)		−9	−61	52	1728	5.03
	RH Precuneus (BA 7)		6	−67	34	81	4.72
	RH Precuneus (BA 31)		6	−49	31	81	3.98
R	Superior frontal gyrus (BA 10)		36	53	13	4590	5.52
	Medial frontal gyrus (BA 10)		15	62	1	81	3.59
R	Middle frontal gyrus (BA 6)		39	8	46	1512	4.68
R	Anterior cingulate (BA 10)		18	38	−8	351	3.86
R	Inferior temporal gyrus (BA 20)		63	−37	−17	432	3.80
R	Inferior parietal lobule (BA 40)		39	−58	43	7614	5.42
	Supramarginal gyrus (BA 40)		48	−43	34	54	3.35
**UNFAMILIAR PROVERBS > FAMILIAR PROVERBS**							
L	Superior frontal gyrus (BA 9)		−9	53	37	1188	4.32
L	Medial frontal gyrus (BA 6)		−9	−4	58	972	4.14
L	Precentral gyrus (BA 4)		−54	−7	46	3321	4.63
L	Middle occipital gyrus (BA 19)		−48	−76	−11	62937	6.21
	Middle temporal gyrus (BA 21)		−57	2	−8	12231	6.07
	Fusiform gyrus (BA 18)		−24	−94	−11	3078	6.03
	Inferior frontal gyrus (BA 47)		−36	29	−26	729	5.54
	Superior temporal gyrus (BA 22)		−51	−13	−5	6831	5.43
	Inferior frontal gyrus (BA 9)		−42	8	22	5373	5.27
	Superior temporal gyrus (BA 22)		−54	−25	1	4914	5.15
	Middle temporal gyrus		−54	−34	−2	54	5.14
	Superior temporal gyrus (BA 38)		−42	20	−17	270	5.05
	Superior temporal gyrus (BA 22)		−51	−37	10	243	4.78
	Middle temporal gyrus (BA 22)		−63	−46	4	108	4.71
	Inferior frontal gyrus (BA 45)		−48	26	7	1836	4.42
	Middle temporal gyrus (BA 22)		−54	−52	4	243	4.34
	Middle temporal gyrus (BA 21)		−39	5	−32	1782	4.25
	Fusiform gyrus (BA 20)		−42	−37	−17	864	4.24
	Parahippocampal gyrus (BA 34)		−30	5	−17	162	3.72
	Cerebellum		−42	−49	−20	189	3.69
	Cuneus (BA 17)		−3	−100	−2	135	3.45
L	Cerebellum		−21	−70	−35	459	4.13
R	Medial frontal gyrus (BA 9)		6	50	34	432	4.12
R	Inferior frontal gyrus (BA 13)		45	26	4	3807	4.60
R	Middle temporal gyrus (BA 21)		51	8	−29	378	3.80
R	Precentral gyrus (BA 6)		57	−7	37	621	3.85
R	Superior temporal gyrus (BA 22)		48	−25	1	6264	4.87
	Superior temporal gyrus (BA 38)		51	8	−14	1134	4.30
R	Inferior occipital gyrus (BA 18)		36	−82	−11	12582	6.02
	Lingual gyrus (BA 18)		24	−94	−5	567	5.81
	Cuneus (BA 17)		3	−100	−2	108	3.62
R	Cerebellum		24	−76	−38	2295	4.24

*Region, sub-region, Talairach coordinates, cluster volume and *Z*-score for the peak voxel of significantly [data corrected to *p* < 0.05 at cluster level, using a height threshold of *p* < 0.005 and a cluster extent threshold determined by individual Monte-Carlo simulations per contrast] activated regions are given for all contrasts related to familiarity differences. Sub-regions larger than 50 mm^3^ are reported if a second peak voxel was found within a different anatomic structure than the peak voxel of the main cluster. Abbreviations: L, Left Hemisphere; R, Right Hemisphere*.

**Table A2:** **Effects of defamiliarization**.

Region	Sub-region	Activation maximum			Volume (mm^3^)	Maximum *Z*-score
		*x*	*y*	*z*
**PROVERB-VARIANTS > FAMILIAR PROVERBS**						
L	Inferior frontal gyrus (BA 44)	−51	14	19	8424	4.55
	Inferior frontal gyrus (BA 45)	−48	29	4	108	4.13
L	Inferior frontal gyrus (BA 47)	−39	17	−17	1431	4.03
	Superior temporal gyrus (BA 38)	−54	8	−11	81	3.18
L	Superior frontal gyrus (BA 6)	−3	8	58	702	3.61
L	Middle temporal gyrus (BA 21)	−54	−52	1	2538	4.17
L	Middle temporal gyrus (BA 21)	−51	−19	−5	297	3.53
L	Precentral gyrus (BA 4)	−51	−4	46	459	3.39
L	Fusiform gyrus (BA 20)	−36	−37	−17	189	3.46
L	Inferior occipital gyrus (BA 17)	−24	−94	−8	3051	4.17
R	Medial frontal gyrus (BA 8)	15	35	43	1404	3.92
	Superior frontal gyrus (BA 8)	9	44	43	81	3.74
	Medial frontal gyrus (BA 9)	6	53	34	81	3.65
R	Inferior frontal gyrus (BA 9)	45	14	19	4725	4.50
	Inferior frontal gyrus (BA 47)	45	29	−5	1944	4.44
R	Inferior frontal gyrus (BA 47)	30	20	−23	324	3.57
R	Superior frontal gyrus (BA 6)	6	8	55	918	4.14
R	Superior temporal gyrus (BA 22)	42	−28	−2	459	3.74
R	Inferior occipital gyrus (BA 17)	24	−97	−11	216	3.39
R	Cerebellum	18	−73	−41	432	3.88
**FAMILIAR PROVERBS > PROVERB-VARIANTS**						
No significant clusters						
**PROVERB-SUBSTITUTIONS > FAMILIAR PROVERBS**						
L	Insula (BA 13)	−45	8	16	2889	4.46
L	Superior temporal gyrus (BA 22)	−51	8	−5	324	3.51
L	Fusiform gyrus (BA 19)	−42	−73	−11	6534	4.01
	Declive	−33	−79	−20	162	3.73
	Inferior occipital gyrus (BA 17)	−24	−100	−11	594	3.71
	Fusiform gyrus (BA 37)	−51	−58	−14	1296	3.66
	Fusiform gyrus (BA 20)	−45	−34	−20	459	3.65
	Tuber	−57	−46	−23	108	3.61
L	Inferior parietal lobule (BA 40)	−51	−37	40	864	3.56
R	Superior frontal gyrus (BA 8)	9	41	52	432	4.15
R	Inferior frontal gyrus (BA 44)	48	11	19	1107	4.10
R	Uncus (BA 36)	18	−4	−35	378	3.97
R	Inferior parietal lobule (BA 40)	45	−46	46	2808	4.45
R	Tuber	45	−52	−23	702	4.12
R	Inferior occipital gyrus (BA 18)	42	−85	−8	459	3.50
**FAMILIAR PROVERBS > PROVERB-SUBSTITUTIONS**						
L	Middle frontal gyrus (BA 8)	−21	26	40	675	4.10
**PROVERB-VARIANTS > PROVERB-SUBSTITUTIONS**						
L	Superior frontal gyrus (BA 8)	−12	47	37	4914	4.91
	Superior frontal gyrus (BA 6)	−15	17	49	1782	4.02
	Superior frontal gyrus (BA 9)	−15	47	22	162	3.78
L	Medial frontal gyrus (BA 11)	0	35	−11	378	3.67
L	Inferior frontal gyrus (BA 47)	−45	26	4	1161	3.58
L	Superior temporal gyrus (BA 38)	−30	14	−20	1161	4.76
L	Superior frontal gyrus (BA 6)	−3	8	58	351	3.39
L	Middle temporal gyrus (BA 21)	−45	5	−29	972	4.19
L	Middle temporal gyrus (BA 21)	−57	−52	4	486	3.45
L	Middle temporal gyrus (BA 39)	−42	−64	25	2808	4.76
	Superior temporal gyrus (BA 39)	−42	−49	22	108	4.17
L	Precentral gyrus (BA 4)	−42	−16	43	621	2.87
L	Postcentral gyrus (BA 3)	−24	−34	49	1215	3.22
L	Posterior parahippocampal gyrus (BA 36)	−24	−31	−11	297	3.16
L	Cuneus (BA 17)	−12	−82	7	324	3.18
L	Inferior occipital gyrus (BA 18)	−24	−88	−8	243	3.05
L	Cerebellum	−3	−55	4	3402	4.20
	Precuneus (BA 23)	0	−58	22	513	4.01
R	Medial frontal gyrus (BA 9)	6	53	34	324	3.65
R	Superior temporal gyrus (BA 38)	33	8	−26	783	3.73
R	Superior frontal gyrus (BA 6)	3	5	58	756	4.17
R	Anterior parahippocampal gyrus/amygdala	21	−13	−11	270	3.08
**PROVERB-SUBSTITUTIONS > PROVERB-VARIANTS**						
L	Anterior cingulate (BA 10)	−18	47	1	864	3.20
L	Fusiform gyrus (BA 20)	−54	−37	−20	486	3.02
L	Cerebellum	−30	−40	−41	1188	3.42
R	Middle frontal gyrus (BA 10)	36	44	10	918	3.82
R	Anterior cingulate (BA 32)	3	35	13	378	2.89
R	Inferior parietal lobule (BA 40)	45	−55	43	378	3.53
R	Cerebellum	30	−28	−32	432	4.34

*Region, sub-region, Talairach coordinates, cluster volume and *Z*-score for the peak voxel of significantly [data corrected to *p* < 0.05 at cluster level, using a height threshold of *p* < 0.005 and a cluster extent threshold determined by individual Monte-Carlo simulations per contrast], activated regions are given for all contrasts related to differences between different types of defamiliarization. Sub-regions larger than 50 mm^3^ are reported if a second peak voxel was found within a different anatomic structure than the peak voxel of the main cluster. Abbreviations: L, Left Hemisphere; R, Right Hemisphere*.
